# Effects of Aerobic Exercise Training on Systemic Biomarkers and Cognition in Late Middle-Aged Adults at Risk for Alzheimer’s Disease

**DOI:** 10.3389/fendo.2021.660181

**Published:** 2021-05-20

**Authors:** Julian M. Gaitán, Hyo Youl Moon, Matthew Stremlau, Dena B. Dubal, Dane B. Cook, Ozioma C. Okonkwo, Henriette van Praag

**Affiliations:** ^1^ Wisconsin Alzheimer’s Disease Research Center and Department of Medicine, University of Wisconsin School of Medicine and Public Health, Madison, WI, United States; ^2^ Lab of Neurosciences, National Institute on Aging (NIA), Baltimore, MD, United States; ^3^ Department of Education, Seoul National University, Seoul, South Korea; ^4^ Institute of Sport Science, Seoul National University, Seoul, South Korea; ^5^ Institute on Aging, Seoul National University, Seoul, South Korea; ^6^ Department of Neurology and Weill Institute for Neurosciences, University of California, San Francisco, San Francisco, CA, United States; ^7^ Department of Kinesiology, University of Wisconsin School of Education, Madison, WI, United States; ^8^ Research Service, William S. Middleton Memorial Veterans Hospital, Madison, WI, United States; ^9^ Geriatric Research Education and Clinical Center, William S. Middleton Memorial Veterans Hospital, Madison, WI, United States; ^10^ Wisconsin Alzheimer’s Institute, University of Wisconsin School of Medicine and Public Health, Madison, WI, United States; ^11^ Brain Institute and Charles E. Schmidt College of Medicine, Florida Atlantic University, Jupiter, FL, United States

**Keywords:** exercise, BDNF, metabolomics, cognition, human, Alzheimer’s disease, Cathepsin B, klotho

## Abstract

Increasing evidence indicates that physical activity and exercise training may delay or prevent the onset of Alzheimer’s disease (AD). However, systemic biomarkers that can measure exercise effects on brain function and that link to relevant metabolic responses are lacking. To begin to address this issue, we utilized blood samples of 23 asymptomatic late middle-aged adults, with familial and genetic risk for AD (mean age 65 years old, 50% female) who underwent 26 weeks of supervised treadmill training. Systemic biomarkers implicated in learning and memory, including the myokine Cathepsin B (CTSB), brain-derived neurotrophic factor (BDNF), and klotho, as well as metabolomics were evaluated. Here we show that aerobic exercise training increases plasma CTSB and that changes in CTSB, but not BDNF or klotho, correlate with cognitive performance. BDNF levels decreased with exercise training. Klotho levels were unchanged by training, but closely associated with change in VO_2_peak. Metabolomic analysis revealed increased levels of polyunsaturated free fatty acids (PUFAs), reductions in ceramides, sphingo- and phospholipids, as well as changes in gut microbiome metabolites and redox homeostasis, with exercise. Multiple metabolites (~30%) correlated with changes in BDNF, but not CSTB or klotho. The positive association between CTSB and cognition, and the modulation of lipid metabolites implicated in dementia, support the beneficial effects of exercise training on brain function. Overall, our analyses indicate metabolic regulation of exercise-induced plasma BDNF changes and provide evidence that CTSB is a marker of cognitive changes in late middle-aged adults at risk for dementia.

## Introduction

Alzheimer’s disease (AD) is the most common neurodegenerative disease. The accumulation of amyloid plaques and neurofibrillary tangles result in a progressive loss of brain function that inevitably leads to mental and physical disability (1). The disease has genetic components, and individuals with a family member affected by AD or who carry the APOE ε4 allele are at increased risk (2). To date there are no effective treatment options for AD patients, and recent pharmacological trials have resulted in failure (3). Therefore, lifestyle interventions such as exercise training that may delay the onset of neurodegenerative conditions have become increasingly imperative (4, 5). In rodents, running training enhances adult hippocampal neurogenesis, synaptic plasticity, and neurotrophin levels throughout the lifespan (4). In aging humans, aerobic exercise training increases gray and white matter volume, enhances blood flow, and improves memory function (5, 6). However, human studies often utilize expensive and low-throughput brain imaging analyses (7, 8) that are not practical for large population-wide studies. Systemic biomarkers that can measure the effect of exercise interventions on AD-related outcomes quickly and at low-cost could be used to inform disease progression and for the development of novel therapeutic targets.

Several exercise biomarkers have been proposed, including growth factor BDNF, myokine CTSB, and klotho. BDNF is an apparent candidate given that this protein is upregulated in the rodent hippocampus and cortex by running and is important for adult neurogenesis, synaptic plasticity, and memory function (9–11). However, the effects of exercise training on human peripheral BDNF levels have been equivocal (5, 12–17). A more recent candidate marker is the myokine (18) CTSB, a lysosomal cysteine protease. Adults with cognitive impairment have lower CTSB levels in serum (19) and brain (20). Several studies indicate CTSB decreases β−amyloid (21, 22), whereas others report that CTSB inhibitors may reduce β−amyloid (23, 24). In young adults, four months of aerobic exercise training elevated plasma CTSB in association with improved fitness and memory function (25). Klotho is another circulating protein that can enhance cognition and synaptic function (26). Klotho is associated with resilience to neurodegenerative disease, possibly by supporting brain structures responsible for memory and learning (27–29). Emerging evidence suggests that its expression is upregulated by exercise (30, 31). Finally, peripheral metabolomic changes associated with dementia have been reported (32–34), but have not been examined in conjunction with an exercise intervention.

In the present study we aimed to determine whether metabolomic profiles related to brain health are beneficially altered following 26 weeks of aerobic exercise training in late middle-aged adults at risk for AD and how such alterations relate to systemic biomarkers such as BDNF, CTSB, and klotho. Therefore, we tested the hypotheses that circulating BDNF, CTSB, and klotho would increase following exercise training and correlate with cognition and metabolomic markers of brain health.

## Materials and Methods

### Characteristics of Study Participants

Participant characteristics have been published previously (35, 36). Briefly, the study enrolled 25 middle-aged adults from the Wisconsin Registry for Alzheimer’s Prevention (WRAP) or the Investigating Memory in People At risk, Causes and Treatments cohort of the Wisconsin Alzheimer’s Disease Research Center (WADRC IMPACT), and 23 completed the trial (see *Results* below). These cohorts are enriched for risk for AD by family history and the APOE ε4 allele. Participants were eligible if they had a family history of AD, were physically inactive [i.e., engaged in less than 150 minutes per week of physical activity (PA)], between 45 – 80 years of age, and cognitively healthy.

### Randomization and Intervention

Details of randomization and study groups have been published elsewhere (35, 36). Briefly, participants were randomized after baseline assessment to either Usual Physical Activity (UPA) or Enhanced Physical Activity (EPA) using 1:1 block randomization accounting for age and sex. The UPA group was given educational material about maintaining a healthy and active lifestyle, with the presumption that they would simply maintain their usual level of PA, i.e., less than 150 minutes per week, during the 26-week study. The EPA group engaged in a supervised, progressive, moderate-to-vigorous intensity aerobic exercise training program three times per week such that from week seven through 26, participants would meet or exceed public health recommendations for PA.

### Cardiorespiratory Fitness Assessment

Details on the cardiorespiratory fitness (VO_2_peak) assessment have been published in detail (35, 36). Briefly, participants completed a graded peak exercise test on a treadmill at a constant self-selected walking speed. The treadmill incline was increased by 2.5% every two minutes until volitional exhaustion. Oxygen consumption, carbon dioxide production, and minute ventilation were recorded using a metabolic cart (TrueOne 2400, Parvomedics, Sandy, UT, USA) after standard calibration procedures to assess VO_2_peak. Criteria for having achieved a VO_2_peak included satisfying at least two of the following: respiratory exchange ratio ≥ 1.10, achievement of 90% of age-predicted maximum heart rate, rating of perceived exertion ≥ 17, and change in VO_2_ < 200 mL with an increase in treadmill incline (37).

### Free-Living Physical Activity Assessment

Details on the assessment of free-living physical activity *via* accelerometry have been published in detail (35, 36). Briefly, participants were fitted with a triaxial accelerometer (GT3X+, Actigraph, Pensacola, FL, USA) that was worn for seven consecutive days during all waking hours except during exposure to water (e.g., bathing). Data inclusion required at least 10 hours of valid wear time per day for a minimum of three weekdays and one weekend day, and data were processed as previously described (36) to yield counts of time spent sedentary, or spent in light, moderate, or vigorous PA.

### Cognitive Function Test

Details of the cognitive function testing have been published previously (35, 36). A trained rater administered a cognitive test battery to each participant. The Mini-Mental State Examination (MMSE) (38) was administered to assess global cognitive function and confirm participants’ cognitively unimpaired status. The California Verbal Learning Test-II (CVLT) (39) was used to assess episodic verbal memory and consists of five learning trials (resulting in the CVLT Total score) and a 20-minute post-learning recall trial (the CVLT Long Delay score). The Delis–Kaplan Executive Function System (D-KEFS) (40) was used to assess executive function. It is comprised of Verbal Fluency, Design Fluency, Trail Making, Sorting, and Color-Word Interference (CWI) subtests. As with our prior publication (36), only the CWI subtest was analyzed for the present report due to its particular sensitivity to executive dysfunction in pre-clinical AD (41). Although the cognitive outcomes analyzed in this study were objective scores (i.e., word counts for CVLT Total and Long Delay scores and time to completion for D-KEFS CWI), the technician responsible for administering cognitive testing was unblinded to treatment allocation.

### Blood Sample Collection

Blood samples were collected after a 12-hour fast from food, caffeine, and alcohol. Samples for plasma analyses were collected into a 10mL EDTA vacutainer which was then gently inverted 10–12 times. Within one hour of collection, EDTA tubes were centrifuged at 3000rpm for 15 minutes at room temperature. Cell-free plasma was then aliquoted into cryovials and stored at –80°C until assayed for CTSB, BDNF, and metabolites. Blood samples for serum analyses were collected into a 9mL vacutainer containing no anticoagulant or additive. The samples were allowed to clot for no more than 30 minutes and were then centrifuged at 3000rpm for 10 minutes at 4°C. Serum was then aliquoted into cryovials and stored at –80°C and was not thawed until assays were run. An additional 4mL whole blood were collected into a 4mL vacutainer containing no additive and was sent to a clinical laboratory to be analyzed for triglycerides, low density lipoprotein (LDL), high density lipoprotein (HDL), and non-HDL cholesterol levels.

### BDNF, CTSB, and Klotho Quantification by Enzyme-Linked Immunosorbent Assay (ELISA)

BDNF and CTSB quantification in plasma was performed using total BDNF ELISA (R&D Systems, Minneapolis, MN, USA) and CTSB ELISA (Abcam, Cambridge, MA, USA) kits, in accordance with the manufacturers’ specifications. Frozen plasma samples were thawed at room temperature (25°C) and held on ice. Samples were loaded in duplicate on a microplate and incubated in accordance with the manufacturers’ protocols. Each assay included recombinant protein as a positive control. Plates were read using SpectraMax Plus 384 Microplate Reader (Molecular Devices, Sunnyvale, CA, USA).

Soluble α-klotho was measured using a solid-phase sandwich ELISA (Immuno-Biological Laboratories, Takasaki, Japan) (42) as described (26, 43). Briefly, serum was diluted four-fold with the immunoassay buffer, a standard curve was created by serial dilution of recombinant human α-klotho protein, and diluted serum was loaded in duplicate onto a plate pre-coated with affinity-purified anti-human klotho (67G3) mouse IgG monoclonal antibody. Control samples were included as references for each plate to enable accurate interplate comparisons. Following incubation for 1 h at room temperature, the plates were washed five times with washing buffer, horseradish peroxidase-conjugated anti-human klotho (100 μl, 91F1) mouse IgG monoclonal antibody was added, plates were incubated for 30 min at room temperature, and then plates were washed five times. The reaction was visualized by addition of 100 μl of chromogenic substrate for 30 min at room temperature and then stopped with 100 μl of 1 *n* H_2_SO_4_. The absorbance at 450 nm was measured on a Spectramax 190 plate reader (Molecular Devices, Sunnyvale, CA, USA), and α-klotho levels were calculated using the SoftMax Pro software (Molecular Devices). Samples with coefficient of variation (CV) above 10% were re-run. Final assay values had an average CV of 2.3% for serum.

### Metabolomics Analysis

#### Sample Preparation for Metabolomic Analysis

Samples were prepared using the automated MicroLab STAR^®^ system (Hamilton Company, Reno, NV, USA). Several recovery standards were added prior to the first step in the extraction process for quality control purposes. Proteins were extracted by precipitation in methanol under vigorous shaking for 2 minutes (Glen Mills GenoGrinder 2000), followed by centrifugation. The resulting extract was divided into five fractions: two for analysis by two separate reverse phase ultrahigh performance liquid chromatography-tandem mass spectroscopy (RP-UPLC-MS/MS) methods with positive ion-mode electrospray ionization (ESI), one for analysis by RP/UPLC-MS/MS with negative ion-mode ESI, one for analysis by hydrophilic interaction chromatography (HILIC)/UPLC-MS/MS with negative ion-mode ESI, and one for potential future analyses. Samples were placed briefly on a TurboVap^®^ (Zymark, USA) to remove organic solvent. Sample extracts were stored overnight under nitrogen before analysis.

#### UPLC-MS/MS

All methods utilized an ACQUITY UPLC apparatus (Waters, Milford, MA, USA) and a Q-Exactive high resolution/accurate mass spectrometer (Thermo Scientific, Waltham, MA, USA) interfaced with a heated ESI source and Orbitrap mass analyzer operated at 35,000 mass resolution. Further details of this analysis are in the Supplementary Information.

### Statistics

Data were analyzed using SPSS 26.0 (IBM Corp., Armonk, NY). Independent samples t-tests (for continuous variables) and Fischer’s exact tests (for categorical variables) were used to analyze baseline differences between treatment groups. Raw metabolite values were normalized in terms of raw area counts by log transformation and rescaled to set the median equal to 1. Missing values, if any, were imputed with the minimum observed value for each metabolite. Two-way repeated measures ANOVA was conducted to test for differences in metabolite levels from baseline to post-intervention by group. Paired samples t-tests were conducted to test differences in metabolites and CTSB, BDNF, and klotho from baseline to post-intervention within each group. Pearson correlations were conducted among systemic biomarkers and cognitive, fitness, physical activity, laboratory, and metabolomic measures.

## Results

### Study Group Participant Properties and Timeline of the Experiments

**Figure 1:**
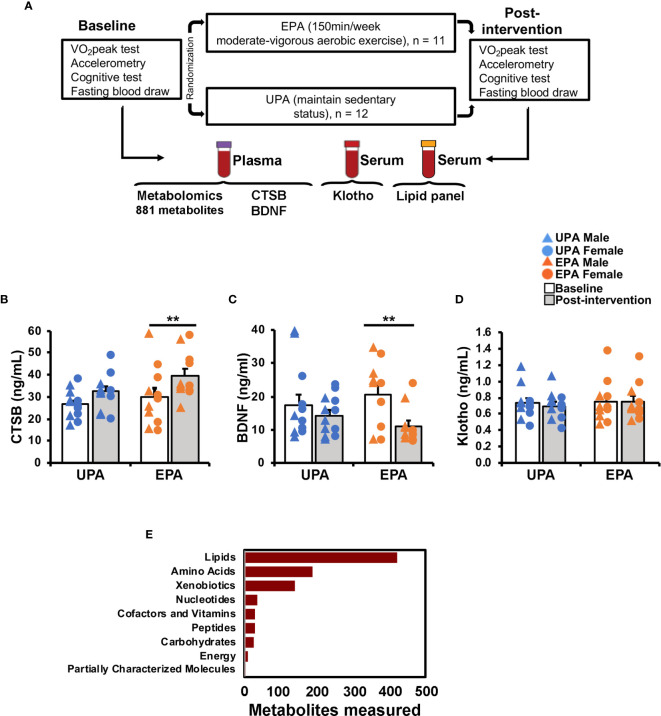
Study design and effects of exercise on circulating biomarkers. **(A)** Overview of study design, exercise training intervention [Usual Physical activity (UPA) or Enhanced Physical Activity (EPA)], and blood sample analyses. The list of outcomes is specific to the present report; other outcomes were measured as part of the trial and are described elsewhere (35). **(B–D)** Effects of the 26-week aerobic exercise intervention on systemic biomarkers. **(B)** Plasma CTSB increased in the EPA group post-intervention as compared to baseline. **(C)** Plasma BNDF decreased in the EPA group. **(D)** Serum klotho levels did not change with exercise. **(E)** Plasma-derived metabolites by super pathway. ** *p* < 0.01 Cathepsin B (CTSB); brain-derived neurotrophic factor (BDNF); Usual Physical Activity (UPA); Enhanced Physical Activity (EPA).

Twenty-five participants enrolled in the study. One participant was excluded after baseline assessment due to an adverse finding on ultrasound imaging, and one discontinued participation in EPA due to an unexpected surgery, resulting in 23 participants completing the study. Participants had a mean age of 64.9 years at baseline. Approximately 50% of the participants were female. On average, participants had 16 years of education, all had parental history of AD, 35% carried at least one copy of the APOE *ε*4 allele, and all were cognitively healthy as further indicated by their baseline MMSE score (mean = 29.61 ± 0.58). There were no significant differences on these baseline characteristics between the groups (**Table 1**). A timeline of the blood sample collection, cognitive testing, and duration of the intervention is depicted in **Figure 1A**.

**Table 1:** Baseline characteristics of the study sample.

	UPA (n=12)	EPA (n=11)	*p*
**Age**	63.92 (5.19)	65.88 (4.00)	0.33
**Female sex, n**	6	5	0.99
**Education**	16.67 (2.57)	16.27 (2.33)	0.71
**Parental history of AD, n**	12	11	–
**APOE ε4 positive, n**	5	3	0.67

Data are mean (standard deviation). Usual Physical Activity (UPA); Enhanced Physical Activity (EPA); Alzheimer’s disease (AD); apolipoprotein E epsilon 4 (APOE ε4).

### Changes in Systemic Biomarkers

Post-intervention CTSB, BDNF, and klotho values in the UPA group were not significantly different compared to baseline levels (CTSB: *t*_(11)_=1.78, *p*=0.102; BDNF: *t*_(11)_=–0.88, *p*=0.396; klotho: *t*_(11)_=1.32, *p*=0.215; **Figures 1B–D**). The EPA group displayed a significant increase in plasma CTSB (*t*_(10)_=3.235, *p*=0.009), reduced BDNF (*t*_(10)_=–3.90, *p*=0.003), and unchanged klotho levels (*t*_(10)_=–0.09, *p*=0.927) after the exercise intervention (**Figures 1B–D**).

### Cardiorespiratory Fitness, Physical Activity, and Cognition Following Exercise Training

Effects of the 26-week exercise training intervention on VO_2_peak, physical activity, sedentariness, and cognition have been published previously (36). Briefly, the EPA group exhibited a greater increase in VO_2_peak and moderate to vigorous physical activity (MVPA), decrease in sedentary behavior, and improvement on the D-KEFS CWI than the UPA group. The improvement on the D-KEFS CWI was correlated with VO_2_peak (36).

### BDNF, CTSB, and Klotho Levels and Cognition

**Figure 2:**
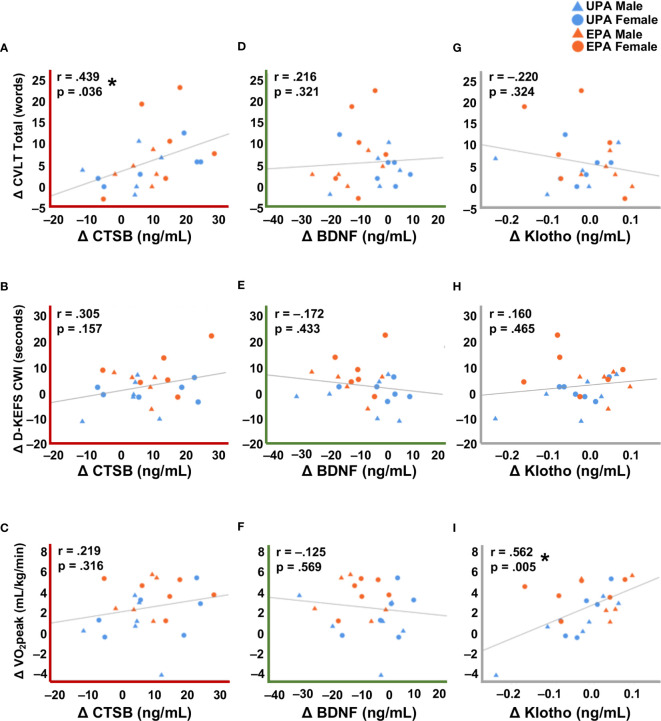
Correlations between changes in systemic biomarkers with cognitive and cardiorespiratory fitness measures. **(A)** Change in CTSB was significantly correlated with verbal learning and memory assessed by the CVLT Total recall score. **(B)** Change in CTSB was correlated, albeit not significantly, with executive function on the D-KEFS CWI. **(C)** There was no correlation between CTSB change and cardiorespiratory fitness. **(D–F)** Change in BDNF was not significantly correlated with cognitive performance or cardiorespiratory fitness. **(G, H)** Change in klotho was not significantly correlated with cognitive performance. **(I)** Change in klotho was significantly correlated with cardiorespiratory fitness. A decrease in the raw D-KEFS CWI scores indicates improvement on this outcome, so D-KEFS CWI scores were inverted such that a positive change indicates improvement in this figure. * *p* <.05. Usual Physical Activity (UPA); Enhanced Physical Activity (EPA); cathepsin B (CTSB); brain-derived neurotrophic factor (BDNF); cardiorespiratory fitness (VO_2_peak); California Verbal Learning Test (CVLT); Delis-Kaplan Executive Function System Color-Word Interference (D-KEFS CWI).

Change in plasma CTSB levels correlated significantly with change in the CVLT Total score (**Figure 2A**, r = .44, *p* <.05) and exhibited a weak but non-significant correlation with the D-KEFS CWI (**Figure 2B**, r = .31, *p* = .16), but not with CVLT Long Delay (**Figure S1A**), VO_2_peak (**Figure 2C**), MVPA, or sedentary behavior (**Table 2**). Change in plasma BDNF was not significantly correlated with any of the cognitive test scores (all r’s <.22, *p*’s >.32**;** **Figures 2D, E** and **S1B**), VO_2_peak (**Figure 2F**), MVPA, or sedentary behavior (**Table 2**). Change in klotho level was significantly correlated with change in VO_2_peak (**Figure 2I**; r = 0.562, *p* = .005; **Table 2**) and exhibited a weak but non-significant correlation with MVPA (r = .33, *p* = .12; **Table 2**) but not with cognitive test scores (**Figures 2G, H** and **S1C**).

**Table 2:** Correlations among cardiorespiratory fitness, physical activity, sedentariness and systemic biomarkers.

	Δ CTSB		Δ BDNF		Δ Klotho	
	r	*p*	r	*p*	r	*p*
**Δ VO_2_peak (mL/kg/min)**	.219	.316	–.125	.569	.562	.005
**Δ MVPA (min)**	–.112	.610	–.275	.204	.332	.122
**Δ Sedentary time (min)**	.035	.875	–.040	.856	–.257	.237

Cathepsin B (CTSB); brain-derived neurotrophic factor (BDNF); Moderate to vigorous physical activity (MVPA).

### Circulating Lipids

Lipid panels were unchanged. There were no significant associations between CTSB, BDNF, or klotho, and blood lipids (all r’s <.36, all *p*’s >.09; **Table 3**).

**Table 3:** Lipid panel results and correlations among lipids and systemic biomarkers.

	UPA (n=12)		EPA (n=11)			Δ CTSB		Δ BDNF		Δ Klotho	
	Baseline	Post-intervention	Baseline		Post-intervention	r	*p*	r	*p*	r	*p*
**Total Chol (mg/dL)**	197.50 (30.16)	186.75 (34.66)	206.00 (39.80)		199.36 (38.14)	.212	.332	.188	.389	.248	.254
**Triglycerides (mg/dL)**	124.75 (50.29)	119.17 (57.35)	114.73 (65.89)		100.64 (58.44)	.247	.255	–.011	.960	–.048	.829
**HDL Chol (mg/dL)**	54.00 (17.37)	50.58 (15.98)	57.73 (17.52)		54.36 (10.71)	–.255	.241	–.115	.602	.364	.087
**LDL Chol (mg/dL)**	118.75 (27.58)	112.42 (28.00)	125.36 (34.72)		124.82 (34.19)	.276	.202	.264	.223	.130	.555
**Non-HDL Chol (mg/dL)**	143.50 (29.76)	136.17 (30.72)	148.27 (34.87)		145.00 (35.97)	.324	.131	.243	.264	.109	.620

Data are mean (standard deviation). Usual Physical Activity (UPA); Enhanced Physical Activity (EPA); cathepsin B (CTSB); brain-derived neurotrophic factor (BDNF); Cholesterol (Chol); high density lipoprotein (HDL); low density lipoprotein (LDL).

### Metabolomic Profiling

For this study, 46 total plasma samples collected at baseline and after 26 weeks of UPA or EPA (post-intervention) were sent to Metabolon for metabolomics analysis (**Figure 1A**). A total of 881 named biochemicals were identified, with lipid metabolites being the most prevalent (**Figure 1E**). Of the 881 total metabolites **(** **Table S1** **),** statistical analyses showed that there was a significant modulation of 78 metabolites (*p*<0.05) in the UPA condition, while in the EPA group there was a significant change in 115 metabolites (*p*<0.05) from baseline to post-intervention. Changes in lipid metabolites accounted for ~50% of total significantly modulated metabolites in EPA [57 (*p*<0.05)] and ~35% (non-overlapping) in UPA [28 (*p*<0.05)]. We focus on 40 lipid metabolites clustered in pathways implicated in AD and exercise (**Table 4**), as well as on 15 metabolites relevant to oxidative stress and the gut microbiome in the EPA group (**Table 5**). Of those 55 metabolites, there was 1 significant correlation with VO_2_peak, 5 with MVPA, 19 with BDNF, 1 with CTSB, and none with klotho **(** **Table 4**; **Table 5** and **Table S2** **).** The entire data set is available upon request and has been deposited in the Metabolomics Workbench, Project ID PR001121 (http://doi.org/10.21228/M8SX23).

**Table 4:** Correlations between BDNF and lipid metabolite changes.

Pathway	Metabolite Name	Δ BDNF		UPA	EPA
		r	*p*	Fold of change	
Lysophospholipid	1-arachidonoyl-GPI (20:4)	–.005	.983	1.15	1.27*
	1-linoleoyl-GPI (18:2)	–.404	.056	1.04	1.37*
	1-oleoyl-GPI (18:1)	–.069	.754	1.14	1.53*
	1-palmitoyl-GPI (16:0)	.001	.998	1.32	1.37†
	1-stearoyl-GPI (18:0)	–.107	.626	1.16	1.46*
	2-palmitoyl-GPC (16:0)‡	–.143	.515	1.02	1.33*
	1-linoleoyl-GPG (18:2)‡	–.078	.725	0.96	1.22†
	1-stearoyl-GPS (18:0)‡	.510	**.013**	0.79	.039*
Phosphatidylethanolamine	1,2-dipalmitoyl-GPE (16:0/16:0)‡	.151	.492	0.86	0.58*
	1-palmitoyl-2-oleoyl-GPE (16:0/18:1)	.352	.099	0.95	0.71*
	1-palmitoyl-2-linoleoyl-GPE (16:0/18:2)	–.073	.740	0.99	0.78*
	1-palmitoyl-2-arachidonoyl-GPE (16:0/20:4)	.503	**.014**	1.08	0.75*
	1-stearoyl-2-oleoyl-GPE (18:0/18:1)	.311	.149	0.85	0.68*
	1-stearoyl-2-linoleoyl-GPE (18:0/18:2)‡	.059	.790	0.95	0.78*
	1-stearoyl-2-arachidonoyl-GPE (18:0/20:4)	.608	**.002**	1.05	0.67*
	1-oleoyl-2-linoleoyl-GPE (18:1/18:2)‡	.102	.645	0.91	0.81*
	1-oleoyl-2-arachidonoyl-GPE (18:1/20:4)‡	.658	**.001**	1.10	0.74*
Phosphatidylinositol	1-palmitoyl-2-linoleoyl-GPI (16:0/18:2)	–.107	.628	0.81	0.76†
	1-palmitoyl-2-arachidonoyl-GPI (16:0/20:4)‡	.028	.898	0.93	0.69*
	1-stearoyl-2-arachidonoyl-GPI (18:0/20:4)	.352	.099	0.98	0.85*
Phosphatidylcholine	1-palmitoyl-2-stearoyl-GPC (16:0/18:0)	.592	.**003**	1.01	0.83*
Polyunsaturated Fatty Acid (n3 & n6)	arachidonate (20:4n6)	.070	.751	1.14	1.27*
	docosahexaenoate (dha; 22:6n3)	–.224	.305	1.12	1.20†
	docosapentaenoate (n6 dpa; 22:5n6)	–.191	.383	1.21	1.38*
	dihomo-linolenate (20:3n3 or n6)	–.074	.736	1.13	1.23*
Ceramides	ceramide (d18:1/20:0,d16:1/22:0, d20:1/18:0)‡	.262	.227	0.93	0.82*
	ceramide (d18:1/14:0, d16:1/16:0)‡	.141	.522	0.91	0.73*
Phospholipid metabolism	choline phosphate	.858	**<.001**	0.87	0.59*
	glycerophosphoethanolamine	.788	**<.001**	0.99	0.77*
	phosphoethanolamine	.854	**<.001**	0.85	0.46*
Dihydrosphingomyelins	sphingomyelin (d18:0/20:0, d16:0/22:0)	.153	.487	0.90	0.71*
	behenoyl dihydrosphingomyelin (d18:0/22:0)	.371	.081	0.85†	0.71*
	palmitoyl dihydrosphingomyelin (d18:0/16:0)‡	.175	.424	0.97	0.88*
Dihydroceramides	n-palmitoyl-sphinganine (d18:0/16:0)	.232	.286	0.91	0.75*
Sphingolipid Synthesis	sphinganine	.724	**<.001**	0.65	0.41*
	sphinganine-1-phosphate	.727	**<.001**	0.84	0.55*
	sphingadienine	.711	**<.001**	0.57	0.28*
Sphingosines	sphingosine	.664	**.001**	0.58	0.34*
	sphingosine 1-phosphate	.668	**<.001**	0.91	0.68*
	hexadecasphingosine (d16:1)‡	.754	**<.001**	0.78	0.44*

Correlations between changes in plasma BDNF levels and lipid metabolites (bolded p values indicate significance). Fold changes of the lipid metabolites in the UPA and EPA groups is listed. ^‡^indicates a metabolite that was identified but has not been confirmed based on a standard of that metabolite. *p < .05. ^†^.05 < p < .10. brain-derived neurotrophic factor (BDNF); Usual Physical Activity (UPA); Enhanced Physical Activity (EPA).

**Table 5:** Correlations between non-lipid metabolites and systemic biomarkers.

Super Pathway	Sub Pathway	Metabolite Name	Δ CTSB		Δ BDNF				Δ Klotho			UPA EPA		
			r	*p*	r		*p*		r	*p*		Fold of change		
**Amino Acid**	Tyrosine Metabolism	3-(4-hydroxyphenyl)lactate	.137	.533	–.262		.228		.086	.695		1.00		1.21*
	Methionine, Cysteine, SAM & Taurine Metabolism	hypotaurine	–.166	.448	.814		**<.001**		.023	.916		0.93		0.51*
		taurine	–.114	.605	.832		**<.001**		.062	.780		0.95		0.63*
		methionine sulfoxide	.031	.888	–.332		.121		.120	.586		0.96		1.14*
	Tryptophan Metabolism	indolepropionate	.487	.**019**	.370		.082		.097	.661		1.10		0.64†
		serotonin	–.234	.282	.413		.**050**		.086	.696		0.21		0.39†
	Urea Cycle; Arginine & Proline Metabolism	n-methylproline	–.018	.935	.018		.936		.116	.599		1.41		1.93*
	Phenylalanine Metabolism	phenylpyruvate	.227	.297	.094		.670		.071	.746		0.87		0.82*
	Polyamine Metabolism	spermidine	–.136	.537	.710		**<.001**		–.028	.901		0.90		0.37*
**Peptide**	Acetylated Peptides	phenylacetylglutamine	–.012	.957	–.196		.371		.222	.309		1.01		1.31*
**Carbohydrate**	Aminosugar Metabolism	glucuronate	.292	.176	.214		.327		.059	.790		1.08		1.91*
		n-acetylneuraminate	.036	.871	.726		**<.001**		.026	.908		0.91		0.76*
**Xenobiotics**	Benzoate Metabolism	3-hydroxyhippurate sulfate	.329	.125	–.007		.976		.120	.584		0.85		1.80*
		3-methoxycatechol sulfate (2)	.055	.803	.257		.237		.323	.133		0.77		0.38*
		o-cresol sulfate	.255	.240	–.231		.288		–.022	.922		1.15		2.43*

Analysis of associations between plasma biomarkers and non-lipid metabolites. Several metabolites display a significant correlation with BDNF (bolded p values). Fold changes of the metabolites in the UPA and EPA groups is listed. Metabolites are classified by Super Pathway and Sub Pathway. *p < .05. ^†^.05 < p < .10. cathepsin B (CTSB); brain-derived neurotrophic factor (BDNF); Usual Physical Activity (UPA); Enhanced Physical Activity (EPA).

#### Changes in Sphingolipid Metabolism

**Figure 3:**
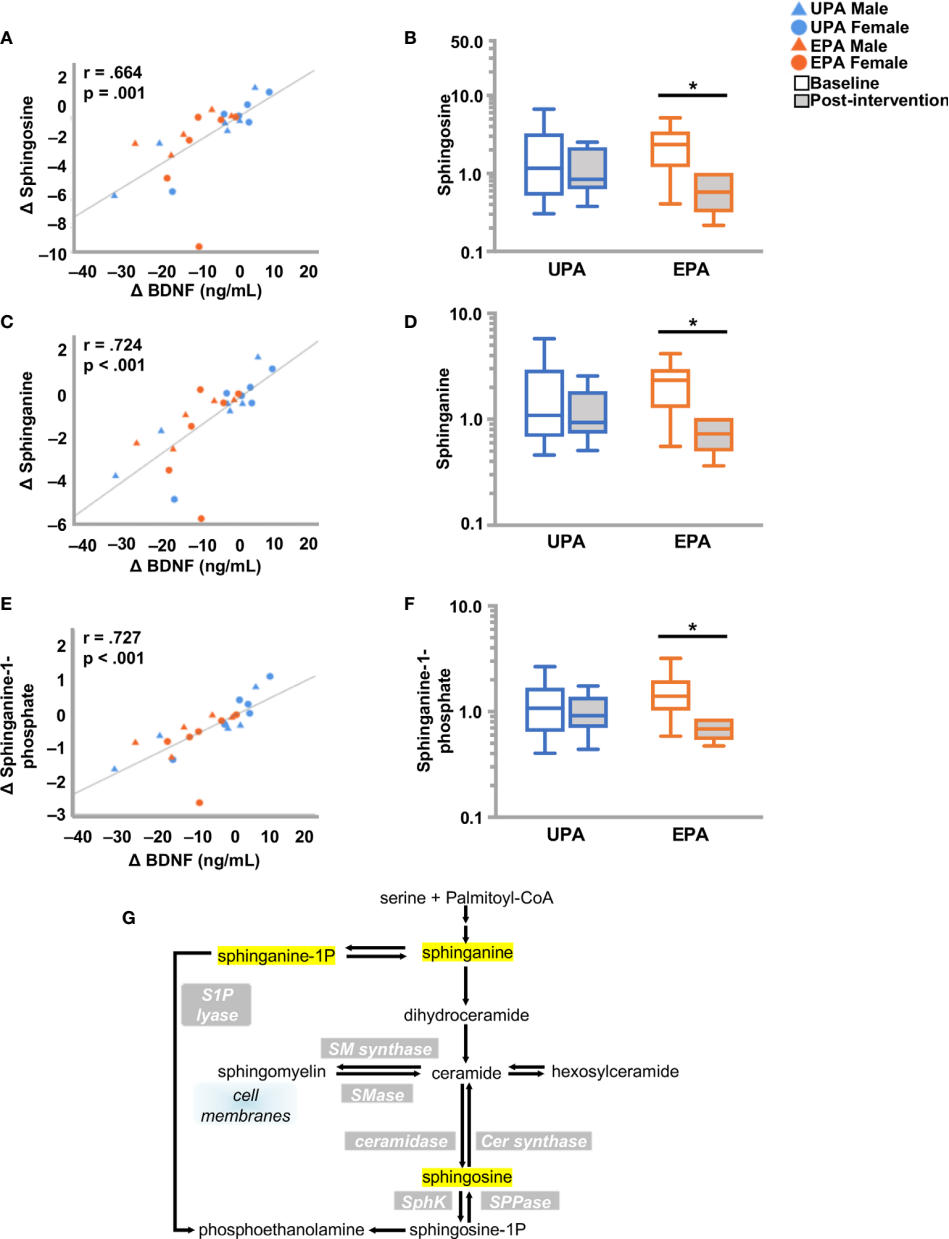
Changes in sphingolipid metabolism and BDNF. **(A, C, E)** Changes in **(A)** sphingosine **(C)** spinganine and **(E)** sphinganine-1-phosphate correlated closely with change in BDNF. **(B, D, F)** Box plots showing levels of these three metabolites were reduced post-intervention in the EPA group. **(G)** Schematic pathway of sphingolipid metabolism. Raw metabolite values were normalized in terms of raw area counts by log transformation and rescaled to set the median equal to 1. *p < .05. brain-derived neurotrophic factor (BDNF); Usual Physical Activity (UPA); Enhanced Physical Activity (EPA).

Sphingolipids are multifunctional lipids, composed of a ceramide (a lipid composed of sphingosine and a fatty acid) and a polar head group (e.g. phosphocholine), that can regulate cell structure (sphingomyelins) and signaling (ceramides, sphingosine-1-phosphate) (44, 45). In the bloodstream, sphingolipids are transported by lipoproteins, primarily by LDL (46). Reductions in several ceramides [e.g., ceramide (d18:1/14:0, d16:1/16:0) and ceramide (d18:1/20:0, d16:1/22:0, d20:1/18:0) and N-palmitoyl-sphinganine (d18:0/16:0)] were observed in EPA post-intervention, as compared to baseline. In addition, dihydrosphingomyelins [sphingomyelin (d18:0/20:0, d16:0/22:0), palmitoyl dihydrosphingomyelin (d18:0/16:0), behenoyl dihydrosphingomyelin (d18:0/22:0)], sphingosines [sphingosine, sphingosine 1-phosphate], and sphingolipid synthesis [sphinganine, sphinganine 1-phosphate, sphingadienine] decreased. Change in sphinganine, sphinganine-1-phosphate, and sphingosine was tightly correlated with plasma BDNF change, **(** **Figure 3** and **Table 4** **)** but not with klotho or CTSB or measures of cognition or VO_2_peak (**Table S2**).

#### Alterations in Phospholipid and Fatty Acid Metabolism

**Figure 4:**
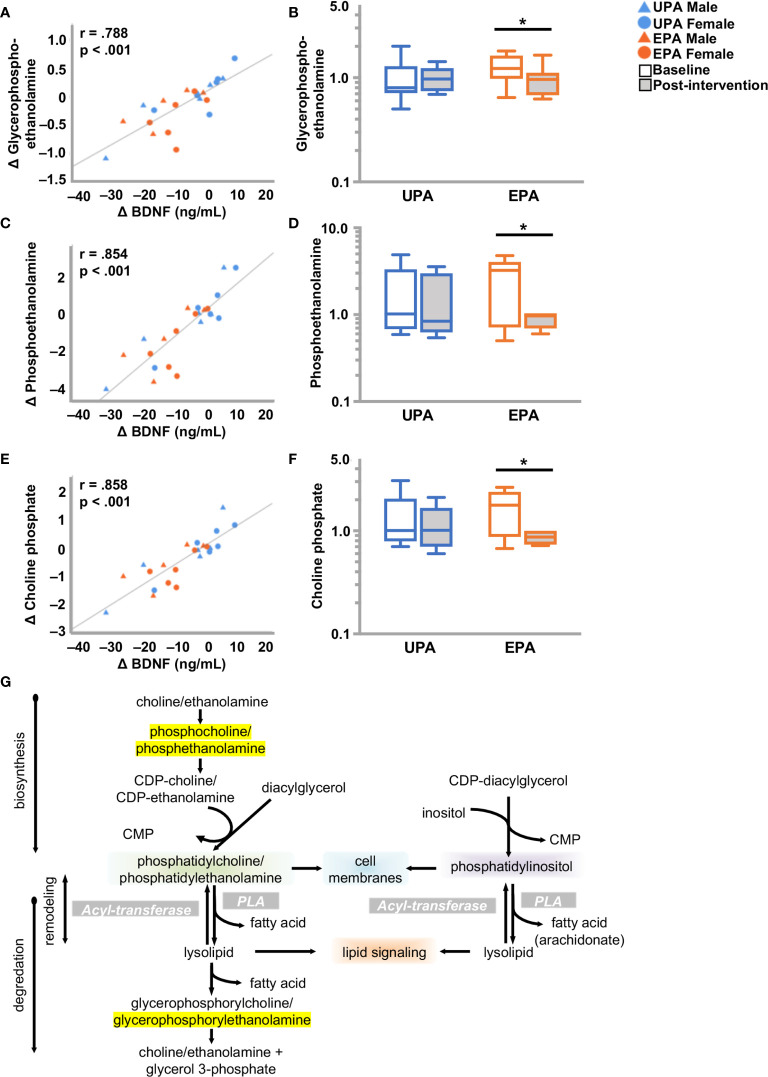
Alterations in phospholipid metabolism and BDNF. **(A, C, E)** Changes in **(A)** glycero-phosphoethanolamine **(C)** phosphoethanolamine and **(E)** choline phosphate correlated closely with change in BDNF. **(B, D, F)** Box plots showing levels of these three metabolites were reduced post-intervention in the EPA group. **(G)** Schematic of the phospholipid metabolic pathway. Raw metabolite values were normalized in terms of raw area counts by log transformation and rescaled to set the median equal to 1. *p < .05. brain-derived neurotrophic factor (BDNF); Usual Physical Activity (UPA); Enhanced Physical Activity (EPA).

Phospholipids are key constituents of the plasma membrane. Phospholipids have a hydrophobic tail consisting of two fatty acids, and a hydrophilic head (e.g., choline, ethanolamine, serine or inositol) to form phosphatidylcholine (PC), -ethanolamine (PE), -serine (PS), or -inositol (PI). Hydrolysis of phospholipids by phospholipase A2 generates free fatty acids (e.g. arachidonate), and 1-lysolipids which are ligands for G-protein coupled receptors that influence development, physiology and disease (47, 48). Post-intervention, the EPA group exhibited notable changes in phospholipid metabolism. Lysophospholipids (7 out of 26 metabolites) were elevated and one metabolite, 1-stearoyl-glycerophophatidylserine (1-stearoyl GPS, 18:0), decreased. Reductions were also observed in one phosphatidylcholine (PC), multiple phosphatidylethanolamines (PE) (9 out of 12 metabolites), and two phosphatidylinositols (PI). In addition, choline increased and choline phosphate (phosphocholine), phosphoethanolamine, and glycerophosphoethanolamine decreased. Changes in 1-stearoyl-GPS (18:0), 1-palmitoyl-2-arachidonoyl-GPI (16:0/20:4), 1-palmitoyl-2-arachidonoyl-GPE (16:0/20:4) 1-stearoyl-2-arachidonoyl-GPE (18:0/20:4), 1-oleoyl-2-arachidonoyl-GPE (18:1/20:4), 1-palmitoyl-2-stearoyl-GPC (16:0/18:0) [also correlated with VO_2_peak, (**Table S2** **)**], glycerophosphoethanolamine, phosphoethanolamine, and choline phosphate correlated closely with change in BDNF, but not with CTSB or klotho (**Figure 4**; **Table 4** and **Table S2**).

Phospholipase activity is also involved in generation of free fatty acids (49). Indeed, the levels of polyunsaturated fatty acids (PUFAs) were increased in the EPA group at post-intervention as compared to baseline, including dihomo-linolenate (20:3n3 or n6), arachidonate (20:4n6), docosapentaenoate (n6 DPA; 22:5n6), and docosahexaenoate (DHA; 22:6n3), while no significant changes were noted in saturated fatty acids (**Table 4** and **Figure S2**).

#### Perturbations in Redox Homeostasis

**Figure 5:**
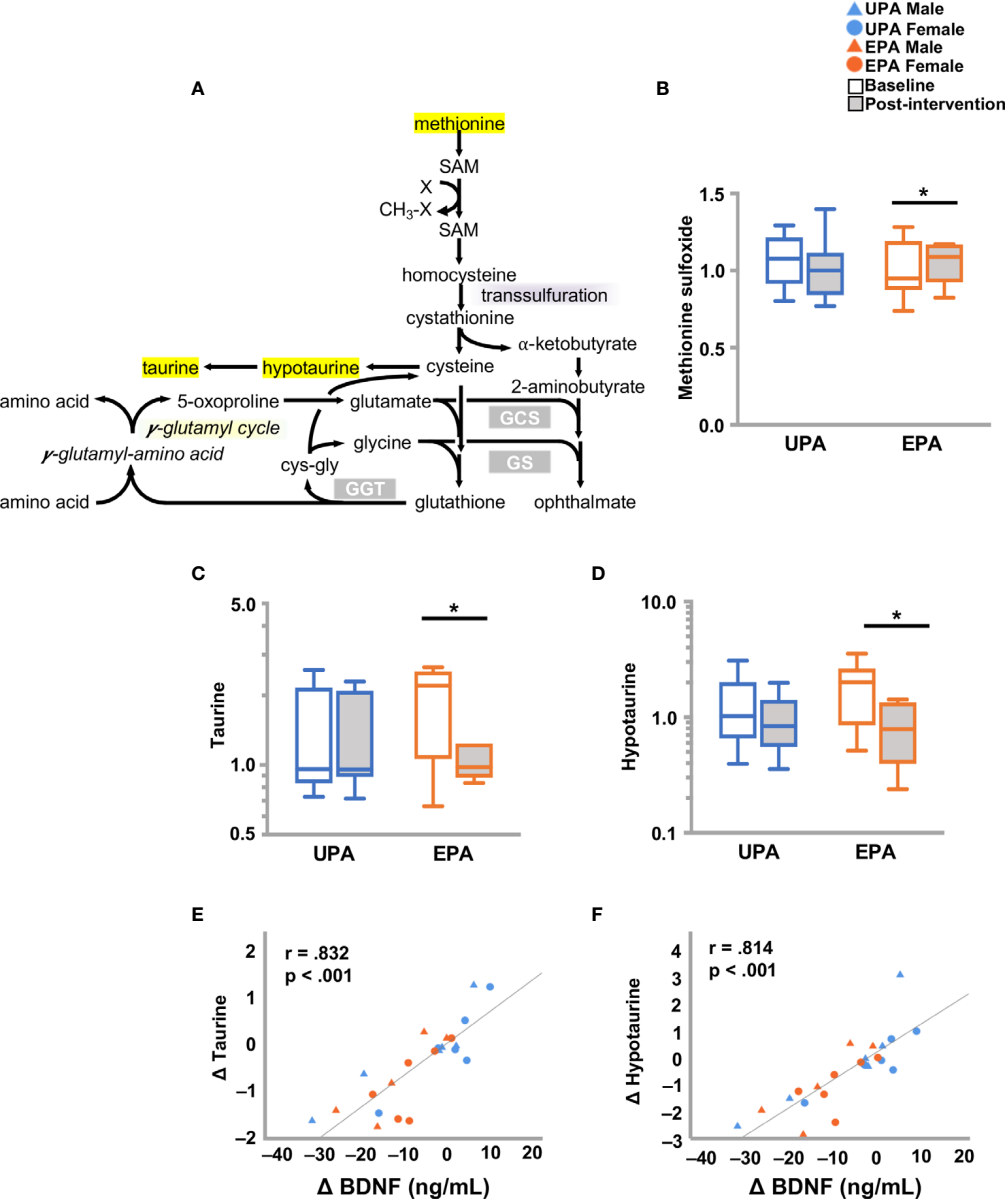
Changes in markers of oxidative stress and BDNF. **(A)** Schematic of the redox pathway. **(B–D)** Exercise training resulted in changes in redox homeostasis with an increase in **(B)** methionine sulfoxide, and decreases in **(C)** taurine and **(D)** hypotaurine. Changes in **(E)** taurine and **(F)** hypotaurine were closely associated with changes in BDNF. Raw metabolite values were normalized in terms of raw area counts by log transformation and rescaled to set the median equal to 1. *p < .05. brain-derived neurotrophic factor (BDNF); Usual Physical Activity (UPA); Enhanced Physical Activity (EPA).

Higher levels of methionine sulfoxide were detected in the EPA group at post-intervention as compared to baseline. In addition, taurine and hypotaurine levels decreased in the EPA group at post-intervention as compared to baseline. Both taurine and hypotaurine change correlated significantly with BDNF change (**Figure 5** and **Table 5**). Furthermore, the levels of the polyamine spermidine (50) were decreased in the EPA group (post-intervention versus baseline). A significant positive correlation was observed between BDNF, but not CTSB or klotho, and spermidine (**Table 5** and **Table S2**).

#### Differences in Molecules Originating in the Gut Microbiome

**Figure 6:**
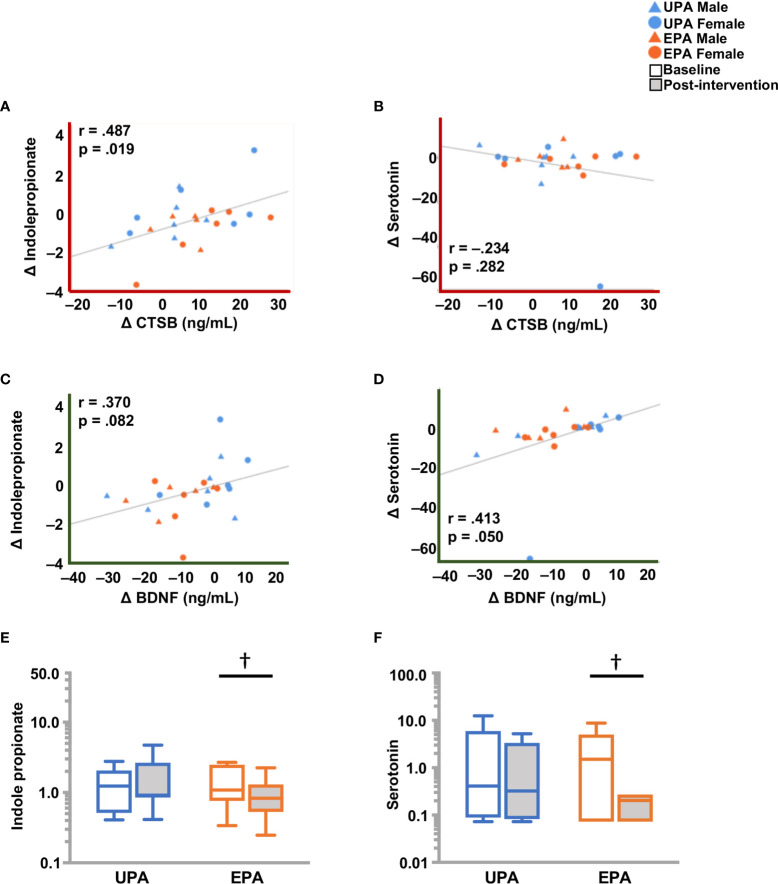
Differences in molecules originating from the gut microbiome, and BDNF and CTSB **(A)** Change in CTSB was significantly associated with a change indoleproprionate (IPA), but not with **(B)** serotonin. **(C)** Change in BDNF showed a weak but non-significant correlation with IPA and **(D)** a significant correlation with serotonin. **(E)** IPA and **(F)** serotonin levels were lower post-intervention in the EPA group. Raw metabolite values were normalized in terms of raw area counts by log transformation and rescaled to set the median equal to 1. ^†^ .05 < *p < .*10. cathepsin B (CTSB); brain-derived neurotrophic factor (BDNF); Usual Physical Activity (UPA); Enhanced Physical Activity (EPA).

Changes from baseline to post-intervention were observed in catabolites of phenylalanine and tyrosine generated by the microbiome. In the EPA group, levels of 3-(4-hydroxyphenyl) lactate and phenylacetylglutamine were higher, while phenylpyruvate levels were lower (**Table 5**). Tryptophan-derived serotonin and indole propionate (IPA) levels also declined in the EPA group at post-intervention as compared to baseline. The change in serotonin was closely linked to BDNF change, whereas IPA was significantly associated with CTSB (**Figure 6**).

Additionally, a number of benzoate-derived compounds were altered over the course of the study. Benzoate metabolites are simple carboxylic acids produced from the microbial degradation of dietary aromatic compounds in the intestine, such as polyphenols (51), purines and aromatic organic acids. 3-hydroxyhippurate sulfate and o-cresol sulfate were elevated in EPA group, whereas 3-methoxycatechol sulfate (2) was reduced at post-intervention as compared to baseline. The metabolite 3-hydroxyhippurate sulfate was the only one evaluated that displayed a positive correlation with cognition (D-KEFS CWI, r = 0.44, *p* = 0.033).

#### Protein Turnover

Higher levels of hydroxyproline (*p* = 0.076) and N-methylproline (*p*<0.05) were detected in the EPA group at post-intervention as compared to baseline. Increased levels of free methylated or hydroxylated amino acids are often indications of enhanced protein degradation, which may reflect changes in muscle composition as a result of physical activity. In particular, hydroxyproline, is specific to the collagen family of proteins (52). Also, changes were noted in aminosugars (e.g., increased glucuronate, decreased N-acetylneuraminate) in the EPA group between post-intervention and baseline. Elevated N-acetylneuraminate has been observed to be increased in the cerebrospinal fluid of AD patients (53). N-acetylneuraminate change was closely correlated with BDNF (**Table 5**).

## Discussion

In absence of therapeutics for AD, exercise has become an important lifestyle factor that may prevent or delay onset of the disease (5, 11). Measuring the effects of exercise on systemic biomarkers associated with risk for AD and relating them to key metabolomic alterations may further prevention, monitoring, and treatment efforts. We evaluated three systemic biomarkers, CTSB, BDNF, and klotho, and performed a metabolomics analysis in the same subjects in blood samples taken at baseline and after a 26-week aerobic intervention. Changes in systemic biomarkers and metabolites were generally consistent with the beneficial effects of exercise for brain function. Plasma CTSB was elevated with EPA and positively associated with cognitive function. Plasma BDNF was diminished in EPA. Serum klotho was unchanged but positively correlated with cardiorespiratory fitness. Metabolomic analysis revealed increased levels of PUFAs and lysophospholipids, and reductions in ceramides, sphingo- and phospho-lipids in EPA. Metabolite levels indicated oxidative stress with EPA; methionine sulfoxide increased and taurine, hypotaurine and spermidine levels decreased. Changes in multiple metabolites correlated closely with BDNF, suggesting that this neurotrophin is regulated by metabolic factors. Overall, the positive correlation between CTSB and cognition, and the substantial modulation of lipid metabolites implicated in dementia, support the beneficial effects of aerobic exercise on brain health in asymptomatic individuals at risk for AD.

Plasma CTSB is upregulated by aerobic exercise training and associated with cognition in young adults (25). In the present study we show that CTSB is increased in late-middle-aged adults after 26 weeks of aerobic training, indicating that CTSB levels change after prolonged activity (25, 54, 55) whereas shorter term aerobic exercise protocols (56, 57), and resistance training (58, 59) show no change. The present findings in late-middle aged adults at risk for AD extend findings from healthy younger adults that CTSB levels were positively correlated with a hippocampus-dependent visual memory task after a four-month aerobic exercise intervention (25). Our results demonstrate that verbal learning and memory, measured by the CVLT, correlated positively with CTSB but was not related to BDNF or klotho. Performance on the CVLT is known to decline in preclinical AD (60). Given that context, the present correlation between CTSB and verbal learning and memory suggests that CTSB may be useful as a marker for cognitive changes relevant to hippocampal function after exercise in a population at risk for AD. In addition, a weak but non-significant positive association between CTSB and the D-KEFS CWI score, an executive function test considered to be mediated by the prefrontal cortex, was observed. A recent study of habitually exercising male adults did not demonstrate an association between CTSB and executive function (61). That study was observational and included participants with decades of exercise training experience who may have long-term adaptation and differences in CTSB signaling not captured by exercise training for 26 weeks in previously sedentary adults. In addition, these subjects participated in a variety of athletic activities. Indeed, older marathon runners have been shown to harbor elevated CTSB levels (55). It is also noteworthy our study included both males and females, and that females may drive the myokine CTSB increase and correlations with cognitive function. A potential underlying mechanism may be muscle physiology differences between females and males (62), but a larger sample would be needed to determine whether sex differences indeed play a role. Our findings help answer a call (63) for data on the effect of exercise-induced CTSB upregulation and cognition, but further studies in males and females are needed to draw stronger conclusions.

While acute exercise generally increases BDNF (15, 57, 64), effects of chronic exercise on blood BDNF levels are less clear (5, 7, 12, 13, 15, 65). In the present study BDNF levels decreased after exercise training and were closely linked to metabolomic alterations. Indeed, in the brain, lipid metabolites (ceramides, sphingolipids) modulate neurotrophin signaling (66–69). However, the regulation of BDNF by metabolites in the blood stream has not been examined under exercise conditions. In our study there was no association between BDNF and verbal learning and memory or executive function. Results of BDNF and cognition following exercise intervention in older adults are mixed. Some reports demonstrate no significant association between BDNF and global cognition (70) or executive function (71), and others indicate increases in both BDNF and cognition among individuals with mild cognitive impairment but do not report associations between the two outcomes (72). In contrast, a recent study demonstrated a significant relationship between plasma BDNF and improved visuospatial ability and verbal fluency, despite no absolute change in resting BDNF levels after three months of aerobic exercise training (73). However, visuospatial ability and verbal fluency are distinct from the cognitive outcomes we measured and may respond differently to BDNF. Moreover, there is some evidence to suggest that BDNF possibly only mediates exercise effects on executive function in adults over the age of 70 (74); our sample had a mean age of approximately 65 years old and thus may not have reached the age threshold needed to realize an effect of BDNF.

There was no change in serum klotho levels after 26 weeks of aerobic exercise training; however, klotho was correlated with increased cardiorespiratory fitness assessed by VO_2_peak, the gold standard. Our finding is consistent with a report that klotho levels showed a weak but non-significant correlation with improvement in a proxy for cardiorespiratory fitness after three months of aerobic exercise in post-menopausal women (75). Other researchers showed that acute bouts of aerobic exercise increase klotho levels in young adults (76) and middle-aged adults of approximately 48 years (31). While older adults respond to acute aerobic exercise with increased klotho levels, the effect is attenuated in adults 65 years and older compared to those aged 25–45 years (77). It is possible that the klotho response to exercise training is similarly blunted among adults 65 years and older. This could explain the lack of change in our sample, as approximately half of our participants were older than 65 years. However, our findings contrast with reports that resting klotho levels increase after exercise training in adults with mean ages in the early 50’s and 60’s (75, 78). It is thus surprising that we did not observe even a modest response. The lack of correlations among klotho and cognitive outcomes in the present study was also surprising given mounting evidence that klotho is associated with multiple cognitive domains including verbal fluency (79), attention, working memory, verbal memory, executive function (80), and global cognition (81) across patient and healthy aging cohorts. However, those associations are primarily based on cross-sectional or longitudinal studies, and our results are interventional. Evidence that augmenting klotho levels can impact cognition in animal models is promising but scarce (82, 83), so further research is needed on whether and how exercise-induced klotho changes in older humans (78) may support cognition.

Metabolomics have become increasingly utilized to understand biochemical pathways that may be affected by AD (32–34). In particular, ceramides, sphingolipids and glycerophospholipids have been proposed as AD biomarkers, and have been linked to cognitive function and mood regulation (33, 84, 85). Ceramides play a role in signaling during apoptosis, inflammation and insulin resistance, processes associated with the pathogenesis of AD (86). Higher ceramide (86, 87) and sphingomyelin (84, 85) levels have been detected in plasma from patients with AD, and depression (88) and have been proposed as diagnostic indicators of these diseases. In the present study, exercise training reduced several ceramide and sphingomyelin species, and dihydrosphingomyelins. This finding is consistent with previous research showing that exercise reduced plasma ceramide (c14:0) in obese and diabetic subjects (89) and in mice (90). Exercise training also decreased sphingosines and sphingolipid synthesis metabolites. The exercise-induced decrease in sphinganine-1-phosphate and precursor sphinganine is of interest, as elevated serum sphinganine-1-phosphate is associated with the conversion from MCI to AD (91). Sphingosine 1-phosphate mediates multiple physiological processes, including regulation of vascular and immune systems and is considered neuroprotective (92–94), albeit that an elevated ratio of d18:1 to d16:1 sphingosine 1-phosphate species may be pro-inflammatory (95). Exercise increased sphingosine 1-phosphate in plasma in young adults (96), but is reduced during recovery (97) and after ultra-endurance races (98). The observed decrease in the EPA group is consistent with other exercise studies (99), however, the functional significance of this reduction remains unclear.

Phospholipids are key constituents of plasma membranes and can contribute to synaptic dysfunction in AD (100). Several of these were changed post-intervention in the EPA group. For instance, choline phosphate is elevated in AD patient cerebrospinal fluid (101) and was lowered in serum after exercise training. In addition, multiple phosphatidylethanolamines, which play a role in inflammation and apoptosis, were reduced by exercise training, consistent with research in mouse models (102). Several lysophospholipids were elevated by exercise training. This finding is of interest since lipids in this pathway are decreased in mild cognitive impairment (MCI) and AD compared to normal controls (103). We also observed that levels of PUFAs were elevated in the EPA group. Exercise training increased omega-6 arachidonate, as well as docosapentaenoate (DPA), an omega-6 considered to be neuroprotective (104, 105), and omega-3 PUFAs docosahexanoate (DHA) and dihomolinolenate (106). In the context of AD, DHA has been shown to reduce both cognitive decline and neuroinflammation (107). It is noteworthy that there were no changes in plasma lipoproteins, such as LDL and HDL cholesterol, which transport phospholipids (46), indicating that exercise specifically induced alterations in lipid metabolite signaling. Furthermore, in the present study no correlations between cognition and changes in lipid metabolites were observed. Both lipid metabolism (108) and BDNF (109) play a role in mood disorders. Multiple phospho-and sphingolipids were tightly associated with change in BDNF levels, suggesting regulation of BDNF levels (110). As depression is linked to AD (111) it would be of interest in further studies to examine the association between mood, exercise, and lipid metabolomics in older adults with AD risk factors.

While the overall effects of exercise in this study appear to be beneficial for brain function, some aspects remain unclear. Aerobic exercise is associated with increased generation of reactive oxygen species, which leads to higher rates of mitochondrial oxidation (112). Increased oxidative stress induced by exercise activates an antioxidant response, which results in elevated expression of antioxidant defense enzymes (113). A redox imbalance (i.e., enhanced oxidant generation and reduced antioxidant defense) has also been associated with neurodegenerative diseases including AD. Accordingly, the dynamic interplay between reactive oxygen species formation and detoxification is involved in maintenance of redox homeostasis. In the present study, we detected higher levels of methionine sulfoxide and lower levels of (hypo) taurine in the EPA group, compared to pre-exercise measurements. Elevated methionine sulfoxide has been reported in people with AD (114), whereas taurine levels are reportedly reduced (115). Taurine exerts neuroprotective functions and acts as an antioxidant. Collectively, differences in redox active compounds that we observed after the 26-week intervention could arise from age- related reductions in antioxidant defense or from the increased risk for AD in these subjects, which can result in oxidative damage of macromolecules. Whether exercise-induced oxidative stress might exert protective effects through the activation of antioxidant response mechanisms in this population remains to be determined. Spermidine is a polyamine metabolite (50) that has been implicated in cognitive function and AD (116, 117) and may alleviate oxidative stress. Increased dietary intake of spermidine is considered beneficial for brain function in people with dementia (116). Consistent with our findings of other antioxidant metabolites, spermidine was diminished in the EPA group.

Physical exercise has been reported to positively affect microbiota diversity and activity, resulting in beneficial health effects (118). Commensal bacteria present in the gut also contribute to the host metabolism by catabolizing and producing a large variety of compounds (119). Changes in the composition of commensal bacteria have been associated with several neurological diseases, including AD (120). We found that exercise increased catabolites of phenylalanine and tyrosine, whereas it reduced levels of serotonin. Although peripheral serotonin is produced in the gut by enterochromaffin cells, gut microbiota can actively modulate the biosynthesis of this neurotransmitter by host cells (121). The reduction in plasma serotonin levels that we detected in the present study is consistent with reported changes in serotonin levels due to exercise (122), and was correlated with the change in plasma BDNF levels (123). Another tryptophan metabolite is indolepropionate (IPA). This was the only metabolite that correlated with change in CTSB. Interestingly, reduced levels, as observed in the present study after exercise, are associated with improved muscle function (124). Elevated levels are associated with inflammation in older adults (125). Collectively, these results support that the gut microbiome is dynamically involved in the anti-depressant effects of physical activity (126) and muscle function.

Several limitations should be considered when interpreting this study. The extent to which these exercise-induced changes in systemic biomarkers reflect alterations in brain function remains unknown, as in-vivo brain measurements post-exercise training are not currently possible. Nonetheless, there is evidence that peripheral BDNF and CTSB influence central levels (25, 127, 128) and that peripheral metabolites such as sphingolipids are associated with AD pathology (33). It is possible that some of our findings are specific to the present study population; comparison to a population without parental history and APOE-related risk for AD would reinforce our conclusions by evaluating whether our findings are specific to those with these particular risk factors. Further research should determine whether the observed systemic biomarker changes also occur in the general population, and larger sample sizes are warranted based on the present findings. This study was designed to measure blood at baseline and post-intervention, so it is possible that acute changes in systemic biomarkers and metabolites (129) occurred with each exercise training session but escaped measurement. Furthermore, the mechanisms underlying metabolite-related changes in plasma BDNF (66–68, 130) with exercise, and whether and how this may result in increased brain levels of this neurotrophin, remains to be elucidated. Moreover, while this study extends and confirms a link between CTSB and cognition, additional older and younger study populations, and memory tests will be needed to further explore this finding. Finally, resistance exercise training may also support brain health (131); however, aerobic exercise was employed in this study due to its upregulation of CTSB and klotho (25, 31, 55), favorable effect on cardiorespiratory fitness (36), and well-established link to cognitive function (5, 6, 132).

In conclusion, plasma CTSB levels were increased following this 26-week structured aerobic exercise training in adults at risk for AD, and change in CTSB was positively associated with cognitive function. Plasma BDNF levels decreased in conjunction with metabolomic changes. Serum klotho was unchanged but was associated with cardiorespiratory fitness. Multiple lipid metabolites relevant to AD were modified by exercise in a manner that may be neuroprotective. Our findings position CTSB, BDNF, and klotho as exercise biomarkers for evaluating the effect of lifestyle interventions on brain function.

## Data Availability Statement

The datasets presented in this study can be found in online repositories. The names of the repository/repositories and accession number(s) can be found below: Metabolomics Workbench, Project ID PR001121 and doi: http://doi.org/10.21228/M8SX23.

## Ethics Statement

The studies involving human participants were reviewed and approved by The University of Wisconsin Institutional Review Board. The patients/participants provided their written informed consent to participate in this study.

## Author Contributions

DC, OO, and HvP designed experiments. JG, HYM, DD, and MS performed experiments and/or analyzed the data. JG, HYM, OO, and HvP drafted the manuscript. All authors revised the manuscript and approved the final version. All authors contributed to the article and approved the submitted version.

## Funding

This work was supported by grants from the Alzheimer’s Association (NIRGD-305257), the Extendicare Foundation, and the National Institutes of Health (K23 AG045957, R01 AG027161, P50 AG033514, and UL1RR025011). This work was also supported in part by the NIA Intramural Research Program, the Florida Atlantic University Brain Institute, Jupiter Life Sciences Initiative, Florida Department of Health, Ed and Ethel Moore Alzheimer’s Disease Research program.

## Conflict of Interest

The authors declare that the research was conducted in the absence of any commercial or financial relationships that could be construed as a potential conflict of interest.
