# Supplementary material for: Effects of Aerobic Exercise Training on Systemic Biomarkers and Cognition in Late Middle-Aged Adults at Risk for Alzheimer’s Disease
Source: Front Endocrinol (Lausanne). 2021 May 20;12:660181. doi: 10.3389/fendo.2021.660181 (PMC8173166; doi:10.3389/fendo.2021.660181)
Supplement: Supplementary file 1 [file DataSheet_1.pdf]

Supplementary Figure 1

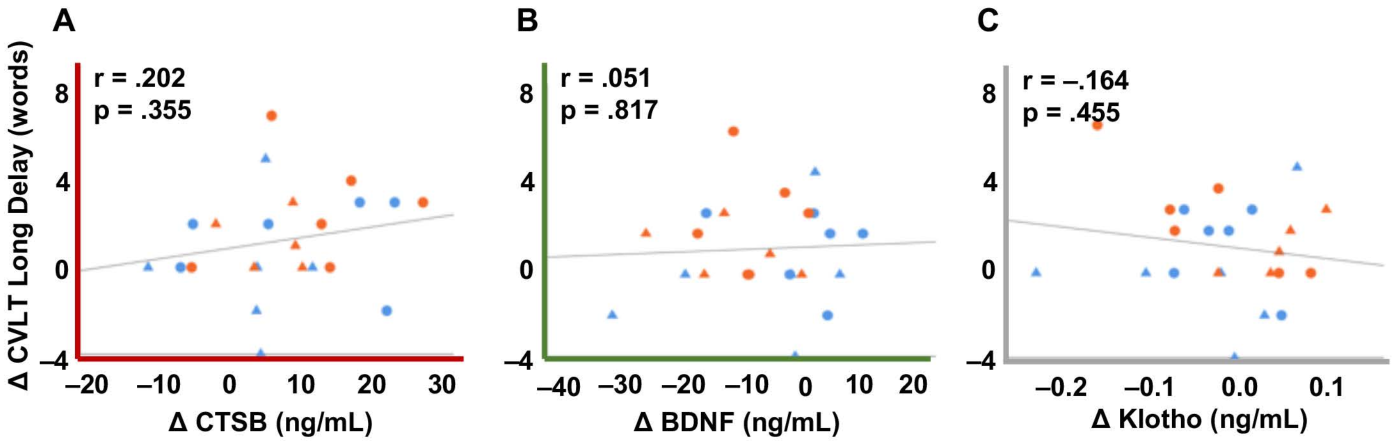

Supplementary Figure 1. Correlations among verbal delayed recall and systemic biomarkers. (A–C) CTSB, BDNF and klotho were not significantly correlated with verbal delayed recall assessed by the CVLT Long Delay. Abbreviations: California Verbal Learning Test (CVLT); cathepsin B (CTSB); brain-derived neurotrophic factor (BDNF); Usual Physical Activity (UPA); Enhanced Physical Activity (EPA).

Supplementary Figure 2

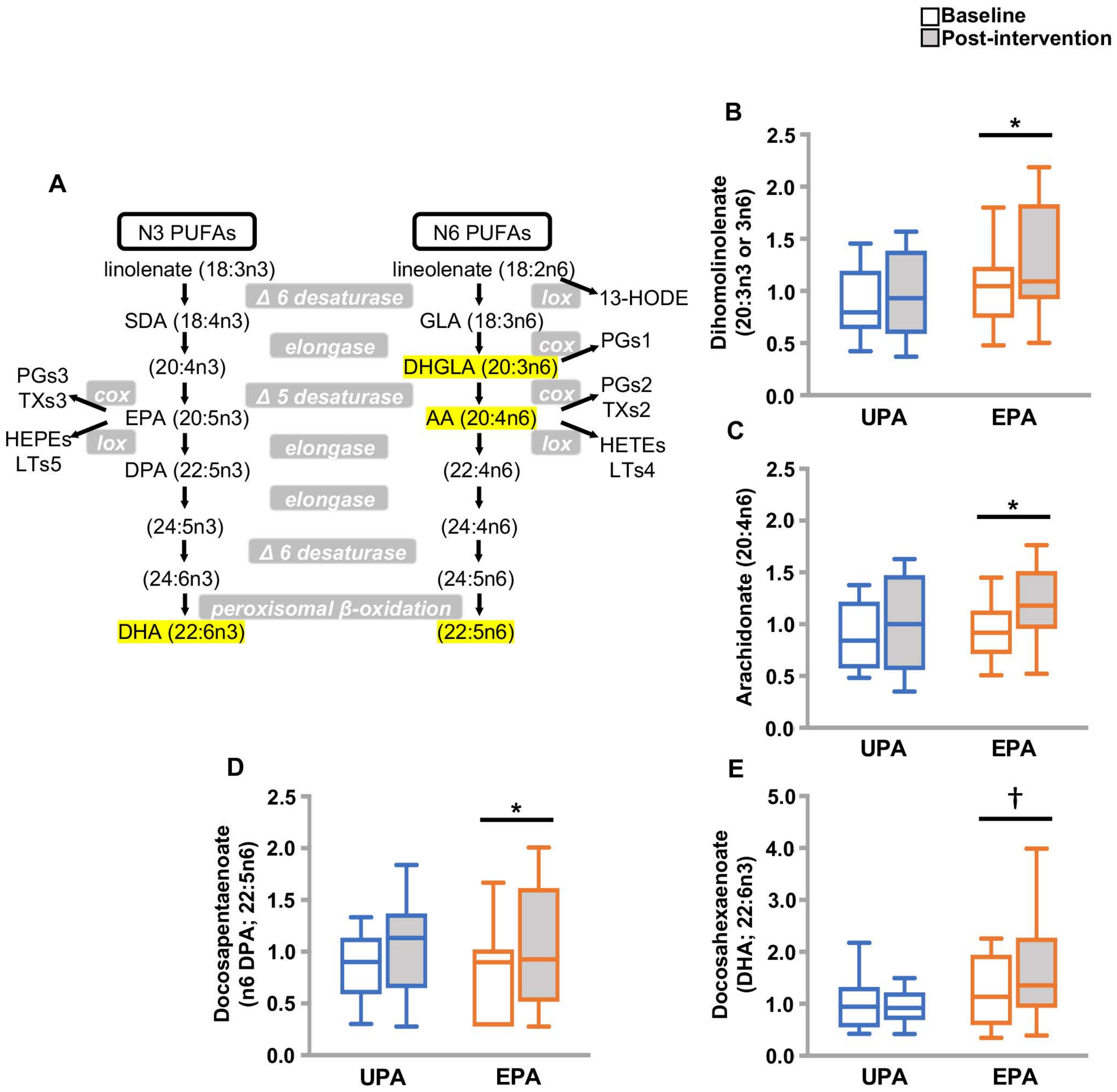

Supplementary Figure 2. Changes in levels of polyunsaturated fatty acids (PUFAs). (A) Schematic of PUFA metabolic pathway. (B) Levels of dihomo-linolenate (20:3n3 or n6), (C) arachidonate (20:4n6), (D) docosapentaoenoate (n6 DPA; 22:5n6), and (E) docosahexaenoate (DHA; 22:6n3) (p<.10) were elevated post-intervention in the EPA group. * $p < .05$. † $.05 < p < .10$. Abbreviations: Usual Physical Activity (UPA); Enhanced Physical Activity (EPA).

Supplemental Tables

Supplemental Table 1. Changes in plasma metabolites

*This table is provided as an Excel file (Table S1_REACH_Metabolite_Heat_Map.xlsx) due to its large size.

All measured metabolites are presented with the fold of change and statistical values for each. In the Fold of Change column: Green indicates $p < .05$ and fold of change < 1.00; Light green indicates $.05 < p < .10$ and fold of change < 1.00; Red indicates $p < .05$ and fold of change ≥ 1.00; Light red indicates $.05 < p < .10$ and fold of change ≥ 1.00.

Supplemental Table 2. Correlations between plasma metabolites and fitness, physical activity, and systemic biomarkers

*This table is provided as an Excel file (TableS2_REACH_Metabolite_Correlations.xlsx) due to its large size.

Metabolites selected for correlation analysis exhibiting significant or nearly significant fold changes in the EPA group. Metabolites are classified by Super Pathway and Sub Pathway. * $p < .05$. † $.05 < p < .10$.

Supplemental Information

Methods

Metabolomic analyses

The sample extract was dried, then reconstituted in solvents compatible with each of the four methods. Each reconstitution solvent contained a series of standards at fixed concentrations to ensure injection and chromatographic consistency. The first aliquot was analyzed using acidic positive-ion conditions and chromatographically optimized for more hydrophilic compounds. In this method, the extract was eluted using a gradient method in a C18 column (Waters, UPLC BEH C18-2.1×100 mm, 1.7 µm) with water and methanol containing 0.05% perfluoropentanoic acid and 0.1% formic acid. The second aliquot was also analyzed using acidic positive-ion conditions; however, it was chromatographically optimized for more hydrophobic compounds. In this method, the extract was eluted using a gradient method in a C18 column with methanol, acetonitrile, water, 0.05% perfluoropentanoic acid, and 0.01% formic acid as solvents; it was operated at an overall higher organic content. The third aliquot was analyzed using basic negative-ion optimized conditions in a separate dedicated C18 column. The basic extracts were eluted using a gradient method from the column in methanol, water, and 6.5 mM ammonium bicarbonate at pH 8. The fourth aliquot was analyzed via negative ionization following elution from a HILIC column (Waters UPLC BEH Amide 2.1×150 mm, 1.7 µm) using a gradient of water and acetonitrile with 10 mM ammonium formate, pH 10.8. MS analysis alternated between MS and data-dependent MSn scans using dynamic exclusion. The scan range varied slightly among methods, but all included 70-1000 m/z. Raw data files were archived and extracted as described below.

Data extraction and compound identification for metabolomic analysis

Raw data were extracted, peak-identified, and processed for quality control using Metabolon hardware and software. These systems are built on a web-service platform utilizing .NET technologies from Microsoft, which run on high-performance application servers and fiber-channel storage arrays in clusters to provide active failover and load-balancing. Compounds were identified after comparison to library

entries of purified standards or recurrent unknown entities. Metabolon maintains a library of molecules based on authenticated standards that contain the retention time/index, mass to charge ratio (m/z), and chromatographic data (including MS/MS spectral data) of all molecules. Furthermore, biochemical identifications were performed based on three criteria: retention index within a narrow retention time/index window of the proposed identification, an accurate match of mass +/- 10 ppm, and the MS/MS forward and reverse scores between experimental data and authentic standards. The MS/MS scores were based on comparisons between ions present in the experimental spectrum and ions present in the library spectrum.

While there may be similarities among molecules based on one of the previously mentioned factors, the use of all three data points further distinguishes and differentiates biochemicals. More than 3300 commercially available purified standard compounds have been acquired and registered into Laboratory Information Management System for analysis on all platforms, with the aim of determining their analytical characteristics. Additional mass spectroscopy entries have been created for structurally unnamed biochemicals, which have been identified because of their recurrent nature (both chromatographic and mass spectrum). These compounds may be identified by future acquisition of a matching purified standard or by classical structural analysis.
